# Authenticity control of pine sylvestris essential oil by chiral gas chromatographic analysis of α-pinene

**DOI:** 10.1038/s41598-021-96356-x

**Published:** 2021-08-19

**Authors:** Martina Allenspach, Claudia Valder, Daniela Flamm, Christian Steuer

**Affiliations:** 1 Institute of Pharmaceutical Sciences, ETH Zürich, 8092 Zurich, Switzerland; 2 Systema Natura GmbH, Konrad-Zuse-Ring 8, 24220 Flintbek, Germany

**Keywords:** Natural variation in plants, Secondary metabolism, Analytical chemistry, Cheminformatics

## Abstract

Numerous terpenes present in essential oils (EOs) display one or more chiral centers. Within the same genus the enantiomeric ratio of these compounds can be different. Thus, the determination of enantiomers is a valuable tool to evaluate authenticity and quality of EOs. In here, the terpene profile of primary and commercial pine EOs was analyzed by conventional and chiral gas chromatography coupled to a flame ionization detector. The enantiomeric excess of ( ±)-α-pinene was determined and significant differences between primary and commercially available EOs were observed. Primary EOs of *Pinus sylvestris* L. showed a positive enantiomeric excess of (+)-α-pinene whereas commercial EOs labeled as *P. sylvestris* L. exhibited an enantiomeric excess of (−)-α-pinene. Thus, chiral analysis provides useful information on the authenticity of pine EOs and allows to uncover possible mislabeling, the use of the wrong herbal substance and sources of adulteration in pine oil.

## Introduction

Essential oils (EOs) defined according to the European Pharmacopeia (Ph. Eur.) are odorous natural products obtained by steam distillation, dry distillation or a suitable mechanical process. Ph. Eur. allows further modifications of an EO, such as rectification^1^. Currently, 34 EOs from different medical plants are described and mono- and sesquiterpenes are the main metabolites found in these herbal preparations. Mono- and sesquiterpenes may show chiral characters. However, a suitable test on chirality is not yet defined for pine EO in the Ph. Eur^1,2^. But determination of chiral characters in a pharmacological active product might be crucial, since enantiomers could differ in their interaction with their biological target^3^. Pine EOs show antibacterial activity i*n vitro* and are used as therapeutic agents in numerous products^4^. As reported previously, (+)-α-pinene and (+)-β-pinene—main constituents of pine Eos—exhibit antimicrobial and anti-inflammatory activity^5,6^. In contrast, negative congeners show insecticidal toxicity^5,7^.

Within the same genus the enantiomeric ratio of main analytes differs from species to species^2,8–10^. In recent years, numerous research groups reported possible chiral impurities in EOs e.g., lavender, bergamot, balm, rose or peppermint EO^2,10,11^. Thus, chiral gas chromatography (GC) analysis is applied to detect the use of the wrong herbal substance or intentional adulteration^12,13^.

According to Ph. Eur, an industrial EO can be deterpenated (removal of monoterpene hydrocarbons), desesquiterpenated (removal of sesquiterpene hydrocarbons), rectified or “x”-free EOs (removal of compound x)^1^. Sesquiterpenes are removed due to their bitter taste and to their potential reduced solubility. During rectification primary EOs are usually re-distilled without water under vacuum to eliminate unwanted compounds with an undesirable odor or with allergic or toxic potential. However, conversion of enantiomeric centers or elimination of enantiomers is not observed^12^.

In order to guarantee appropriate quality of therapeutically used herbal substances the establishment of reliable and traceable qualities becomes more and more important. The supply chain for EOs usually involves many steps during collection of the starting material to the finished distilled product. Possible supply chain issues affect the quality of the final product. Especially, collection activities in non-defined agricultural environment often result in quality issues arising from confusion of closely related medicinal plants^12,14^. Chiral analysis therefore offers a unique tool to verify potential treatment other than that defined in the Ph. Eur. during supply chain assessment.

The total evaluation of a correct supply chain including unchangeable analytical markers as well as traceable information on the exact origin, harvest and storage of materials as defined in the good agricultural and collection practices (GACP)^12^ are therefore a perfect combination in the frame of a successful herbal material qualification.

In case of EOs of *Pinus sylvestris* L. (*P. sylvestris*) three registered suppliers (European Chemicals Agency [ECHA]) import the EOs from different locations worldwide to provide them to local purchasers^15^.

Previously, our group investigated the chromatographic profiles of primary EOs of *P. sylvestris* obtained from traceable authentic plant material and developed a partial least squares discriminant analysis (PLS-DA) model for the correct taxonomic classification of closely related pine species^4^. The present work evaluates the chiral gas chromatographic-flame ionization detector (GC-FID) profile of primary and commercially available pine EOs as additional tool for authenticity control. Our results clearly demonstrate that for comprehensive quality control the enantiomeric ratio of at least the major terpenes in pine EOs is of high importance.

## Results and discussion

**Figure 1:**
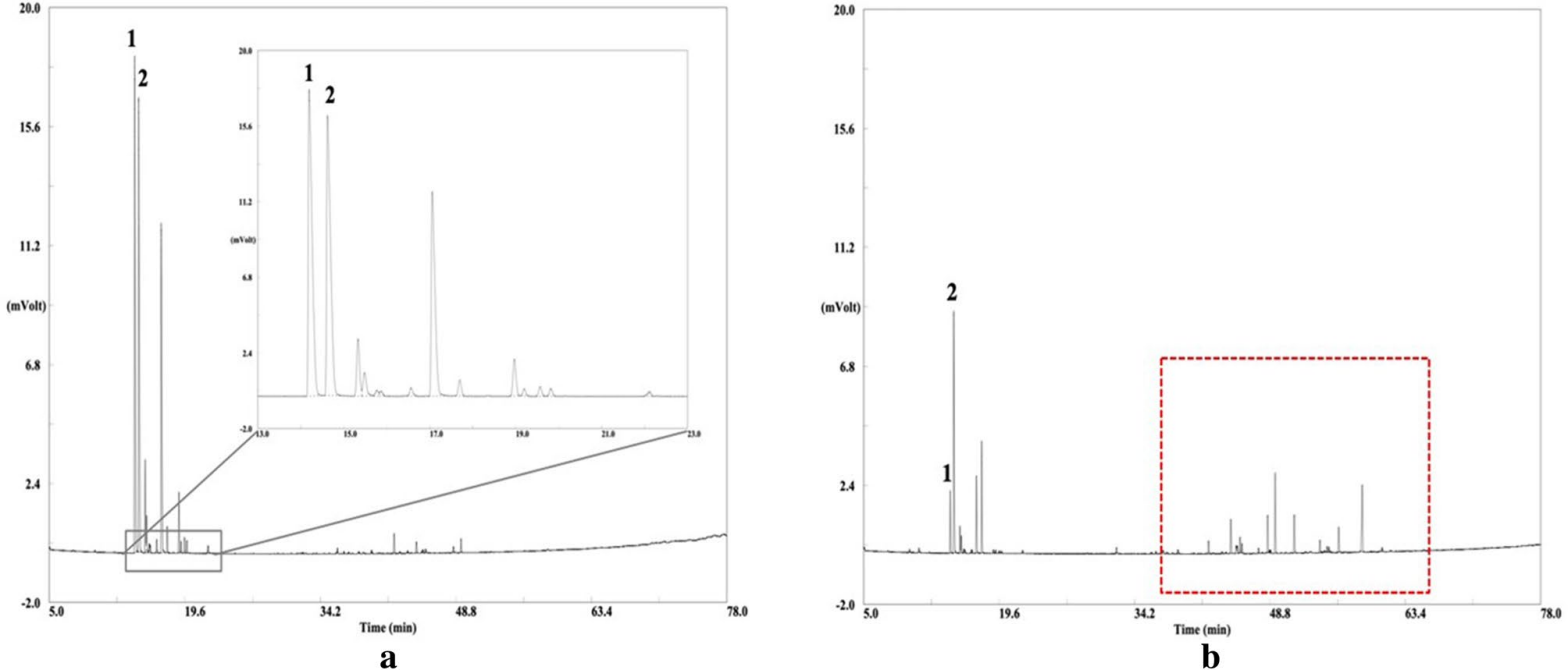
(**a**) Chromatographic profile obtained by chiral gas chromatography-flame ionization detector of a commercial essential oil of *P. sylvestris* (**1**) and (**b**). primary essential oil of *P. sylvestris* (**36**) with (−)-α-pinene (**1**, RT: 14.3 min, RI cal: 977, RI lit: 977) and (+)-α-pinene (**2**, RT: 14.7 min, RI cal: 983, RI lit: 980). The red square indicates the distinct sesquiterpene profile, which was lacking in commercial EOs.

Commercial EOs of *P. sylvestris* were obtained from the three registered suppliers for *P. sylvestris* EO (**1**–**4**, European Chemicals Agency [ECHA], Supplementary Information, Table A1)^15^ and local providers, respectively. In general, the chromatographic profile of a primary pine EOs from assigned and traceable pine trees (Supplementary Information, Table A2) consists of monoterpenes, sesquiterpenes and their oxygenated derivatives^4^. All commercial essential oils fulfilled the criteria given by the Ph. Eur. for pine sylvestris oil. Our results clearly indicated, that commercial EOs were predominantly composed of monoterpene hydrocarbons with α-pinene as dominant analyte. However, commercial EOs of *P. sylvestris* showed significant different sesquiterpene pattern (Fig. 1a, Supplementary Information, Table A3) compared to primary EOs of the corresponding species (Fig. 1b, Supplementary Information, Table A4). A possible reason for the reduced sesquiterpene profile could have been modification of primary EOs e.g. by rectification^1^. One may speculate, if the low amount of minor compounds is the result of further dilution. Dilution with turpentine oil or turpentine oil based substances is a well-known adulteration in pine EOs^12,16^.

**Table 1:** Taxonomic classification of commercial essential oils of *P. sylvestris* by the developed PLS-DA model^4^ and the enantiomeric excess of (−)-α-pinene.

Commercial EOs of *P. sylvestris*	Predicted class	Enantiomer	Enantiomeric excess (%)
1	*P. nigra*	(−)-α-pinene	3.1 ± 0.0
2	Not classified	(−)-α-pinene	19.8 ± 0.0
3	Not classified	(−)-α-pinene	78.4 ± 0.1
4	*P. nigra*	(−)-α-pinene	76.0 ± 0.0
5	Not classified	(−)-α-pinene	74.3 ± 0.0
6	Not classified	(−)-α-pinene	82.4 ± 0.0
7	Not classified	(−)-α-pinene	69.1 ± 0.0
8	*P. nigra*	(−)-α-pinene	76.1 ± 0.1
9	*P. nigra*	(−)-α-pinene	66.7 ± 0.1
10	*P. nigra*	(−)-α-pinene	8.6 ± 0.1
11	Not classified	(−)-α-pinene	83.0 ± 0.0

Data were expressed as mean ± standard deviation (*n* = 3).

**Figure 2:**
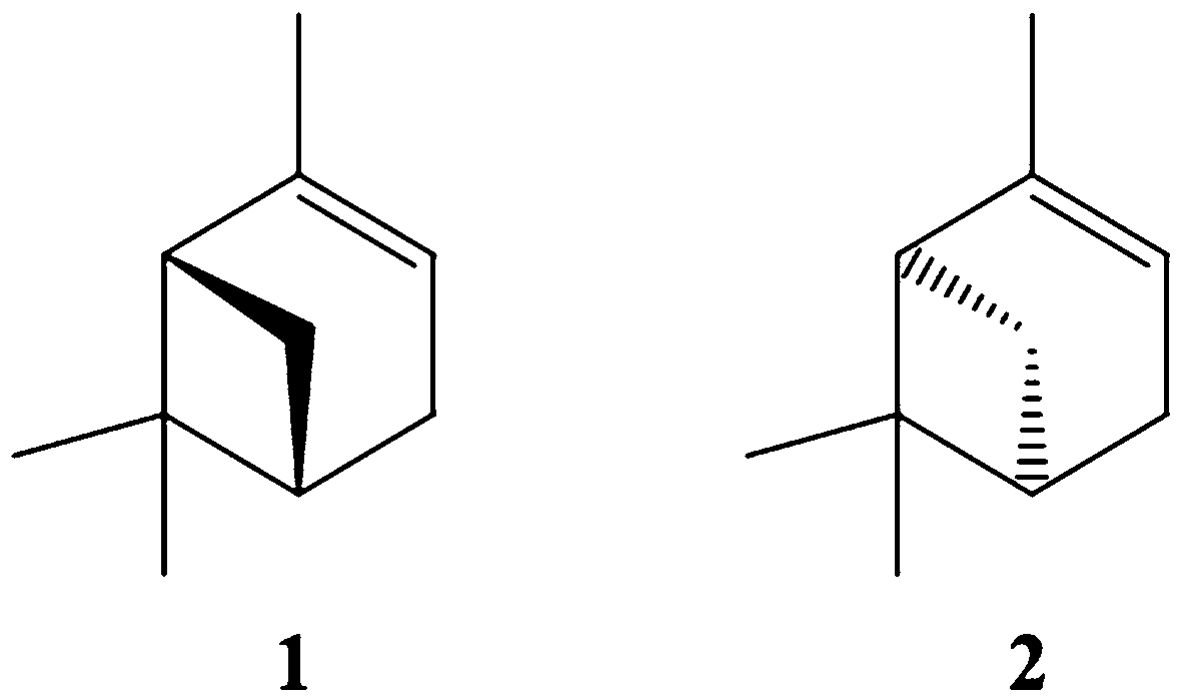
Chemical structures of the two enantiomers: (−)-α-pinene (**1**) and (+)-α-pinene (**2**).

As illustrated in Table 1, the previously developed PLS-DA model was applied for taxonomic identification of commercial EOs of *P. sylvestris*^4^. Although they were labeled as *P.* sylvestris, none of them were classified as *P. sylvestris.* The commercial EOs remained either unclassified (*n* = 6) or were classified as EO derived from *P. nigra* (*n* = 5). Subsequently, an additional analytical tool is required to detect possible adulterations. Thus, all commercial and primary pine essential oils were analyzed by chiral GC-FID. Enantiomers of α-pinene (Fig. 2) were baseline separated with a resolution of R_s_ = 2.1 (Fig. 1a). In commercial and primary pine EOs, α-pinene was present in both enantiomeric forms (+/−).

**Figure 3:**
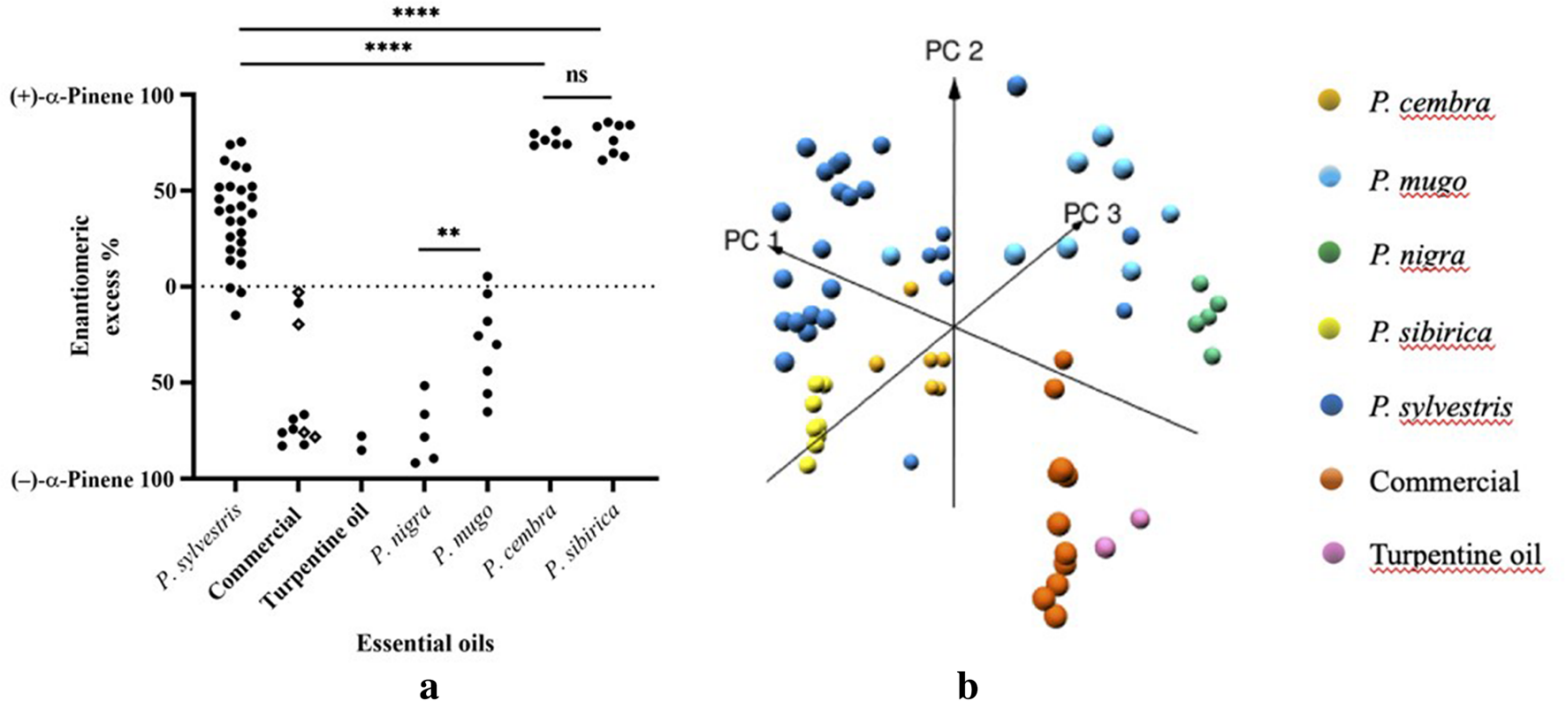
(**a**) The enantiomeric excess values (%) of (±)-α-pinene of primary, commercial pine EOs (ECHA products in diamond shape) and turpentine oil. Significance of the enantiomeric excess values (%) of (+)-α-pinene and (−)-α-pinene were tested using Welch’s ANOVA test followed by Games-Howell’s multiple comparisons post-hoc test with *p* < 0.01 (**), *p* < 0.0001 (****) and ns: not significant. (**b**) The 3D score plot of the first three principal components PC1 (45.5%), PC2 (15.2%) and PC3 (9.5%) for primary pine and commercial EOs based on their chemical composition obtained by the conventional GC-FID and ( ±)-α-pinene. EOs are colored based on their taxonomic specification and commercial origin, respectively.

The dominant enantiomer in *P. sylvestris* of different origin was (+)-α-pinene (Fig. 3a). Until recently, only one research group reported an excess of (+)-α-pinene in EOs derived from *P. sylvestris*^17^. *P. cembra* showed the same dominant enantiomer as *P. sylvestris* (significantly different with *p* < 0.0001: ****) whereas the enantiomer (−)-α-pinene was predominant in *P. nigra* and *P. mugo* (significantly different with *p* < 0.01: **), commercial EOs and turpentine oils. Only in three *P. silvestris* samples, a minor excess of (−)-α-pinene was detected. Our preliminary results showed that primary pine EOs were classified into their taxonomic specification by PLS-DA, a supervised chemometric method^4^. However, adding chiral information of α-pinene to the existing 39 variables and fourth root calculation as data preprocessing, unsupervised principle component analysis (PCA) was sufficient to uncover data patterns in GC-FID chromatograms. Three-dimensional (3D) PCA allowed a clear taxonomic separation between primary pine and commercial EOs (Fig. 3b) but also in-between primary oils of different pines based on the first three principal components (PC1, PC2 and PC3) explaining 70.2% of the total variance. However, PCA revealed chemical similarity of commercial EOs and turpentine oils emphasizing possible dilution with turpentine oil based substances. The corresponding biplot is presented in the Supplementary Information, Figure A1.

The majority of the commercial EOs showed the opposite enantiomeric ratio of (±)-α-pinene compared to primary EOs of *P. sylvestris* and exhibited a high ee of (−)-α-pinene (Fig. 3a). This is in-line with a previous report, which exhibited the enantiomeric content in commercially manufactured pine EOs^18^. Our data showed, that the commercial EOs were distinguished into two groups. Most of the commercial EOs belonged to group 1 (65.0–85.0%) whereas the ee of the second group ranged from 0.0–20.0%. Although the second group showed similar ee values as observed for primary EO´s the main part of all commercial oils is significant different regarding the ee. According to our data, a threshold of e.g. 17.5% ee for (−)-α-pinene would allow identification of a commercial essential oil (specificity: 1.0; sensitivity: 0.82). Additionally, commercial EOs could be assigned to one of the three registrants in terms of their ee of (−)-α-pinene (Fig. 3a). Finally, the therapeutic benefit of these EOs is questionable, since (+)-α-pinene is responsible for the antimicrobial and anti-inflammatory effects, but they predominantly consisted of (−)-α-pinene^5,6^.

A possible reason for the high ee of (−)-α-pinene could have been an intended or unintended confusion in the raw material. Since *P. nigra* shares the same habitat and shows similar morphological properties to *P. sylvestris*. Lack of botanical knowledge could have led to misidentification and confusion in the raw material. Obviously, commercial EOs showed a similar enantiomeric pattern compared to turpentine oil. In turpentine oil—byproduct of paper industry—, also an excess of (−)-α-pinene was found (Fig. 3a)^19^. Based on our results, we are convinced that chiral analysis is helpful to uncover possible mislabeling and sources of adulteration in pine essential oils. Additionally, enantiomeric distribution of (±)-α-pinene can be used to assign the commercial EOs of second suppliers to one of the three registered ECHA-distributors. Another example reinforced chiral analysis as an additional tool to determine authenticity of primary EOs. Primary EOs (**60**–**67**), labeled as *P. sylvestris*, remained unclassified and exhibited a high ee of (+)-α-pinene (Fig. 3a). Considering their Siberian origin, they were assigned as *P. sibirica,* known as a subspecies of *P. cembra*. EOs of *P. sibirica* and *P. cembra* were not significantly different in terms of their ee of (+)-α-pinene (*p* > 0.05, ns). However, difference to *P. sylvestris* was highly significant (*p* < 0.0001: ****).

To ensure the correct identification of pine species used as starting material international standards are recommended. Good Agricultural and Collection Practice for medicinal and aromatic plants (GACP) provides unique guidelines on correct collection of raw material. GACP controls the quality during cultivation, harvesting, processing, labeling and storage to obtain herbal products of correctly identified authentic plants for therapeutic and recreational use^12,20^.

In conclusion, for the evaluation of the correct herbal substance and preparation in the field of pine EOs—next to a traceable supply chain—the unique chromatographic quality including analytical markers is crucial. Chromatographic profile obtained by GC-FID is able to distinguish between primary and commercial pine EOs. Lacking sesquiterpene patterns in further treated commercial oils might be explained by rectification. Chiral analysis however provides additional significant information on the authenticity of pine EOs and allows to uncover possible mislabeling, the use of wrong herbal substances and/or adulteration within the supply chain.

## Materials and methods

### Material

Commercially available EOs of *P. sylvestris* (*n* = 11, **1**–**11**) and turpentine oils (*n* = 2, **12**–**13**) were purchased from different suppliers. A detailed overview and used in-house codes are given in the Supplementary Information, Table A1. Primary Pine EOs were obtained from needles and twigs collected from *P. sylvestris* (*n* = 27, **14**–**40**), *Pinus cembra* L. (*P. cembra*) (*n* = 6, **41**–**46**), *Pinus mugo* TURRA (*P. mugo*) (*n* = 8, **47**–**54)**, *Pinus nigra* J. F. ARNOLD (*P. nigra*) (*n* = 5, **55**–**59**) and *Pinus sibirica* DU TOUR (*P. sibirica*) (*n* = 8, **60**–**67**). Plant material was classified by macroscopic botanical identification. A detailed overview of the used in-house codes, GPS coordinates and harvesting times can be found in the Supplementary Information, Table A2. The EO of freshly cut (pieces of 1 cm) needles and twigs was obtained by industrial distillation.

### GC-FID analysis

The GC-FID analysis was performed using a Thermo Fisher Scientific Focus gas chromatograph (Thermo Fisher Scientific, Waltham, Massachusetts, USA) equipped with a DB-wax capillary column (30 m × 0.25 mm i.d., film thickness 0.25 μm). The temperature of the injection was 220 °C. The injection volume was 1 μl (Autosampler AI3000, Thermo Fisher Scientific) using a split ratio of 1:50 with a split flow of 75 ml/min. Helium was used as carrier gas at a constant flow rate of 1.5 ml/min. The oven temperature was kept at 65 °C for 10 min and then heated to 220 °C with 5 °C/min and kept constant at 220 °C for 9 min. The temperature of the detector was 250 °C. Primary EOs diluted in heptane and commercial EOs (ten-fold diluted with heptane) were analyzed using the relative percentages of the individual components based on the FID response (peak area). The data were acquired with Chrom Card Trace Focus GC (Thermo Fisher scientific, version 2.9).

The chiral GC-FID analysis (Thermo Fisher Scientific, Waltham, Massachusetts, USA) was performed using a Thermo Fisher Scientific Trace 1300 gas chromatograph equipped with a BGB 176 SE capillary column (30 m × 0.25 mm i.d., film thickness 0.25 μm). The chiral column consists of 30% 2,3-dimethyl-6-*tert*-butyldimethylsilyl-β-cyclodextrin dissolved in SE-52 (5% phenyl-, 95% methylpolysiloxane). The temperature of the injection was 220 °C. The injection volume was 1 μl (commercial EOs) or 2 μl (primary EOs) (Autosampler AI3000, Thermo Fisher Scientific) using a split ratio of 1:70 with a split flow of 28 ml/min. Helium was used as carrier gas at a constant flow rate of 2.5 ml/min. The oven temperature was kept at 50 °C for 3 min and then heated to 200 °C with 2 °C/min. The temperature of the detector was 250 °C. Peaks were identified by comparing retention times (RT) with reference substances. (−)-α-Pinene was purchased from Fluka Chemie GmbH (Buchs, Switzerland). (+)-α-Pinene was obtained from Sigma-Aldrich (St. Louis, MO, USA). Retention indices (RI cal) were calculated according to the van den Dool and Kratz equation with the RI in the literature (RI lit)^21,22^.

20 μl of the EOs was diluted with heptane to 10 ml and the enantiomeric excess (ee) in % was calculated from peak area (PA) by the following Eq. (1). The data were acquired with Chrom Card Trace Focus GC (Thermo Fisher scientific, version 2.9).

1 $$ ee\, \left( \% \right) = \frac{PA\,of\,predominant\,enantiomer - PA\,of\,minor\,enantiomer}{{PA\,of\,predominant\,enantiomer + PA\,of\,minor\,enantiomer}} \times 100 $$

### Statistical analysis

The statistical analysis and illustration were carried out using GraphPad Prism 8 (version 8.0.0 (224)) software. Results were expressed as mean ± standard deviation. Mean values were compared by either unpaired t-test (with Welch’s correction when no homoscedasticity) or an ordinary one-way Welch’s ANOVA test (with Welch’s correction when no homoscedasticity) followed by Games-Howell’s multiple comparisons post-hoc test. A *p* value < 0.05 was considered to be statistically significant. Prior to ANOVA, normal distribution using Shapiro–Wilk test (α = 0.05) and homoscedasticity using Brown-Forsythe test (*p* < 0.05) were asserted. ChemDraw Professional (version 19.0.0.26) was used to generate the chemical structures. Principle component analysis (PCA) was performed on fourth root calculated data. The dataset was composed of the commercial (*n* = 11) and primary pine EOs (*n* = 54) characterized by 39 compounds and ( ±)-α-pinene (Supplementary Information, Table A3 and A4). PCA was performed with Rstudio (version 1.2.5019; packages: ggbiplot, version 0.55; pca3d, version 0.10).

## Supplementary Information

Supplementary Information.
