# Supplementary material for: Authenticity control of pine sylvestris essential oil by chiral gas chromatographic analysis of α-pinene
Source: Sci Rep. 2021 Aug 19;11:16923. doi: 10.1038/s41598-021-96356-x (PMC8376981; doi:10.1038/s41598-021-96356-x)
Supplement: Supplementary file 1 — Supplementary Information. [file 41598_2021_96356_MOESM1_ESM.docx]

Supplementary Information

**Authenticity control of pine sylvestris essential oil by chiral gas chromatographic analysis of α-pinene**

**Martina Allenspach^1^, Claudia Valder^2^, Daniela Flamm^2^, Christian Steuer^1,*^**

^1^Institute of Pharmaceutical Sciences, ETH Zürich, 8092 Zürich, Switzerland

^2^Systema Natura GmbH, Konrad-Zuse-Ring 8, 24220 Flintbek, Germany

*[christian.steuer@pharma.ethz.ch](mailto:christian.steuer@pharma.ethz.ch)

|  | **Name** | **Charge** | **Supplier** |
| --- | --- | --- | --- |
| 1 | Pin sylvestre | 181828 | Aroma Plantes (Sault, France) |
| 2 | Kiefernadel, Pinus sylvestris | 281-679-2 | Brüder Unterweger GmbH (Thal-Aue, Austria) |
| 3 | Kiefernnadelöl, natürlich | - | Frey + Lau (Henstedt-Ulzburg, Germany) |
| 4 | Kiefernnadelöl | S0100060 | Frey + Lau |
| 5 | Waldkiefer | 765035-712 | Aromasan (Cugy, Switzerland) |
| 6 | Kiefernnadel | V29R01 | Bergland Pharma GmbH & Co. KG (Heimertingen, Germany) |
| 7 | Kiefernadel Alpen | 709 | Elixan Aromatica GmbH (Gähwil, Switzerland) |
| 8 | Pini silvestris aetheroleum | 2018.12.0174 | Haenseler (Herisau, Switzerland) |
| 9 | Miaroma Pine 100% pure EO | B60190 | Holland & Barrett International Limited, Nuneaton, United Kingdom |
| 10 | Pin sylvestre Bio | A4351/3 | Nabio (Vevey, Switzerland) |
| 11 | Waldkiefer | 765027-475 | Aromasan |
|  | Pin sylvestre | P204288 | De Saint Hilaire (Saint-Hilaire, France) |
| 12 | Terpentinöl, China | S0401993 | Frey + Lau GmbH |
| 13 | Terpentinöl, Portugal | S0100138 | Frey + Lau GmbH |

**Table A1.** Code, name, charge and supplier of the commercial EOs of *P. sylvestris.*

| **EO** | **Species** | **Country** | **GPS coordinates** | **Harvesting time** | **PLS-DA model** |
| --- | --- | --- | --- | --- | --- |
| 14 | *Pinus sylvestris* L. | Canada | - | - | PS |
| 15 | *Pinus sylvestris* L. | Denmark | - | August 2019 | Predicted as PS |
| 16 | *Pinus sylvestris* L. | Denmark | - | August 2019 | PS |
| 17 | *Pinus sylvestris* L. | Germany | N47° 40’ 40’’  E9° 10’ 23’’ | December 2019 | PS |
| 18 | *Pinus sylvestris* L. | Germany | N47° 40’ 40’’  E9° 10’ 23’’ | December 2019 | PS |
| 19 | *Pinus sylvestris* L. | Germany | N47° 40’ 40’’  E9° 10’ 23’’ | December 2019 | Predicted as PS |
| 20 | *Pinus sylvestris* L. | Germany | N47° 40’ 40’’  E9° 10’ 23’’ | December 2019 | PS |
| 21 | *Pinus sylvestris* L. | Germany | N47° 40’ 40’’  E9° 10’ 23’’ | December 2019 | PS |
| 22 | *Pinus sylvestris* L. | Germany | N48° 07’ 56’’  E11° 34’ 21’’ | February 2020 | Predicted as PS |
| 23 | *Pinus sylvestris* L. | Germany | N48° 07’ 56’’  E11° 34’ 21’’ | February 2020 | Predicted as PS |
| 24 | *Pinus sylvestris* L. | Germany | N48° 07’ 56’’  E11° 34’ 21’’ | February 2020 | Predicted as PS |
| 25 | *Pinus sylvestris* L. | Germany | - | January 2020 | Predicted as PS |
| 26 | *Pinus sylvestris* L. | Germany | - | January 2020 | Predicted as PS |
| 27 | *Pinus sylvestris* L. | Germany | N54° 19’ 16’’  E10° 8’ 05’’ | - | Predicted as PS |
| 28 | *Pinus sylvestris* L. | Russia | - | Winter 2018 | PS |
| 29 | *Pinus sylvestris* L. | Russia | - | Winter 2018 | PS |
| 30 | *Pinus sylvestris* L. | Russia | - | Winter 2018 | PS |
| 31 | *Pinus sylvestris* L. | Russia | - | - | Predicted as PS |
| 32 | *Pinus sylvestris* L. | Russia | - | - | Predicted as PS |
| 33 | *Pinus sylvestris* L. | Russia | - | - | Predicted as PS |
| 34 | *Pinus sylvestris* L. | Russia | - | - | Predicted as PS |
| 35 | *Pinus sylvestris* L. | Russia | - | - | Predicted as PS |
| 36 | *Pinus sylvestris* L. | Italy | N46° 26’ 08’’  E11° 17’ 37’’ | September 2020 | PS |
| 37 | *Pinus sylvestris* L. | Switzerland | N47° 25’ 16’’  E9° 16’ 31’’ | August 2019 | PS |
| 38 | *Pinus sylvestris* L. | Switzerland | N47° 24’ 29’’  E8° 30’ 24’’ | July 2019  December 2019 | PS |
| 39 | *Pinus sylvestris* L. | Switzerland | N47° 23’ 54’’  E8° 32’ 36’’ | August 2019 | PS |
| 40 | *Pinus sylvestris* L. | Switzerland | N47° 27’ 35’’  E9° 31’ 28’’ | August 2019 | PS |

**Table A2.** Origin data of the primary EOs (-: no information about the location provided) and their classification according to the PLS-DA model developed in Allenspach, M., Valder, C., Flamm, D., Grisoni, F. & Steuer, C. Verification of chromatographic profile of primary essential oil of *Pinus sylvestris* L. combined with chemometric analysis. *Molecules* **25**, 2973, doi:10.3390/molecules25132973 (2020).

|  | **Species** | **Country** | **GPS coordinates** | **Harvesting time** | **PLS-DA model** |
| --- | --- | --- | --- | --- | --- |
| 41 | *Pinus cembra* L. | Switzerland | N46° 27’ 35’’  E9° 47’ 45’’ | November 2019 | PC |
| 42 | *Pinus cembra* L. | Switzerland | N46° 27’ 35’’  E9° 47’ 45’’ | November 2019 | PC |
| 43 | *Pinus cembra* L. | Switzerland | N46° 27’ 35’’  E9° 47’ 45’’ | November 2019 | PC |
| 44 | *Pinus cembra* L. | Switzerland | N46° 29’ 23’’  E9° 54’ 15’’ | November 2019 | PC |
| 45 | *Pinus cembra* L. | Switzerland | N46° 25’ 45’’  E9° 45’ 49’’ | November 2019 | PC |
| 46 | *Pinus cembra* L. | Switzerland | N46° 06’ 28’’  E7° 55’ 38’’ | December 2019 | PC |
| 47 | *Pinus mugo* TURRA | Switzerland | N47° 17’ 03’’  E9° 24’ 39’’ | August 2019 | PMu |
| 48 | *Pinus mugo* TURRA | Denmark | - | August 2019 | PMu |
| 49 | *Pinus mugo* TURRA | Switzerland | N46° 29’ 45’’  E9° 50’ 19’’ | November 2019 | PMu |
| 50 | *Pinus mugo* TURRA | Switzerland | N46° 29’ 45’’  E9° 50’ 19’’ | November 2019 | PMu |
| 51 | *Pinus mugo* TURRA | Germany | N47° 40’ 40’’  E9° 10’ 23’’ | December 2019 | PMu |
| 52 | *Pinus mugo* TURRA | Switzerland | N47° 08’ 22’’  E8° 32’ 09’’ | February 2020 | PMu |
| 53 | *Pinus mugo* TURRA | Switzerland | N47° 08’ 22’’  E8° 32’ 09’’ | February 2020 | PMu |
| 54 | *Pinus mugo* TURRA | Switzerland | N47° 08’ 22’’  E8° 32’ 09’’ | February 2020 | PMu |
| 55 | *Pinus nigra* J. F. ARNOLD | Switzerland | N47° 25’ 31’’  E9° 15’ 32’’ | November 2019 | PN |
| 56 | *Pinus nigra* J. F. ARNOLD | Switzerland | N47° 24’ 23’’  E9° 20’ 25’’ | November 2019 | PN |
| 57 | *Pinus nigra* J. F. ARNOLD | Germany | N49° 23’ 34’’  E7° 3’ 38’’ | December 2019 | PN |
| 58 | *Pinus nigra* J. F. ARNOLD | Switzerland | N47° 24’ 29’’  E8° 30’ 24’’  E8° 30’ 24’’ | February 2020 | PN |
| 59 | *Pinus nigra* J. F. ARNOLD | Switzerland | N47° 24’ 29’’  E8° 30’ 24’’ | February 2020 | PN |
| 60 | *Pinus sibirica* DU TOUR | Russia | - | - | Unclassified |
| 61 | *Pinus sibirica* DU TOUR | Russia | - | - | Unclassified |
| 62 | *Pinus sibirica* DU TOUR | Russia | - | - | Unclassified |
| 63 | *Pinus sibirica* DU TOUR | Russia | - | - | Unclassified |
| 64 | *Pinus sibirica* DU TOUR | Russia | - | - | Unclassified |
| 65 | *Pinus sibirica* DU TOUR | Russia | - | - | Unclassified |
| 66 | *Pinus sibirica* DU TOUR | Russia | - | - | Unclassified |
| 67 | *Pinus sibirica* DU TOUR | Russia | - | - | Unclassified |

**Table A2.** (continued).

| **Compounds** | **1** | **2** | **3** | **4** | **5** | **6** | **7** | **8** | **9** | **10** | **11** | **12** | **13** |
| --- | --- | --- | --- | --- | --- | --- | --- | --- | --- | --- | --- | --- | --- |
| Tricyclene | 0.4 | - | 0.7 | - | 0.2 | 0.1 | 0.1 | 0.1 | 0.1 | 0.5 | 0.1 | 0.1 | 0.1 |
| α-Pinene | 59.9 | 45.7 | 29.9 | 44.5 | 62.3 | 61.9 | 44.8 | 35.5 | 46.4 | 43.1 | 56.6 | 80.7 | 71.0 |
| Camphene | 2.4 | 4.6 | 9.3 | 1.4 | 1.6 | 1.6 | 1.0 | 1.1 | 1.3 | 2.5 | 1.6 | 1.7 | 0.9 |
| β-Pinene | 19.8 | 22.1 | 6.5 | 21.1 | 10.5 | 10.0 | 20.4 | 20.8 | 21.4 | 38.4 | 10.5 | 6.3 | 15.7 |
| Sabinene | 0.1 | 0.1 | 0.2 | - | - | - | - | 0.1 | - | 0.1 | - | - | - |
| 3-Carene | 1.3 | 1.6 | 16.7 | 12.8 | 6.4 | 6.5 | 8.4 | 15.6 | 13.7 | 0.1 | 5.9 | 0.4 | - |
| β-Myrcene | 4.4 | 5.6 | 4.3 | 0.9 | 2.1 | 1.5 | 0.5 | 1.9 | 1.9 | 1.2 | 1.0 | 0.3 | 0.4 |
| Limonene | 3.3 | 9.6 | 11.3 | 9.5 | 7.6 | 8.2 | 8.7 | 10.2 | 7.1 | 5.0 | 7.9 | 1.6 | 3.3 |
| β-Phellandrene | 0.8 | 2.2 | 0.2 | 0.4 | 0.1 | 0.1 | - | 0.9 | 0.3 | 0.5 | - | 0.3 | 0.2 |
| p-Cymene | 0.6 | 1.3 | 0.9 | 1.8 | 1.5 | 1.6 | 1.9 | - | 1.8 | 0.2 | 1.6 | 1.7 | 0.2 |
| Terpinolene | 0.3 | 0.5 | 3.0 | - | - | - | - | 3.0 | 0.1 | 0.2 | - | 0.2 | 0.1 |
| Bornyl acetate | 0.1 | 1.0 | 8.1 | 1.7 | 0.6 | 0.7 | 2.1 | 3.1 | 1.0 | 0.1 | 1.1 | - | 0.1 |
| α-Terpineol | 0.3 | 0.6 | 1.0 | - | 0.4 | 0.2 | - | 0.5 | 0.3 | 0.6 | 0.3 | 0.5 | 0.7 |
| Longipinene | - | - | - | - | - | - | - | - | - | - | - | - | - |
| Copaene | 0.1 | 0.1 | 0.1 | - | - | 0.1 | 0.1 | - | 0.1 | - | 0.1 | - | 0.2 |
| Longifolene | 0.2 | 0.1 | 0.7 | 1.2 | 0.6 | 0.5 | 1.5 | 0.5 | 0.4 | 0.2 | 1.1 | 2.0 | 1.8 |
| β-Caryophyllene | 1.1 | 0.9 | 2.3 | 0.5 | 0.1 |  |  | 3.7 | 0.4 | 1.0 | - | 0.4 | 1.4 |
| Guaia-6,9-diene | 0.3 | - | - | - | - | - | - | - | - | - | - | - | - |
| α-Humulene | 0.2 | 0.2 | 0.2 | 0.1 | - | - | - | 0.5 | 0.1 | 0.1 | - | 0.1 | 0.2 |
| γ-Muurulene | 0.2 | 0.1 | - | - | - | - | - | - | - | - | - | - | - |
| Germacrene d | 0.7 | - | - | - | - | - | - | - | - | - | - | - | - |
| β-Selinene | - | 0.1 | - | - | - | - | - | - | - | - | - | - | - |
| α-Selinene | - | 0.1 | - | - | - | - | - | - | - | - | - | - | - |
| α-Muurolene | 0.2 | 0.2 | - | - | - | - | - | - | - | - | - | - | - |
| Bicyclogermacrene | 0.2 | 0.1 | - | - | - | - | - | - | - | - | - | - | - |
| γ-Cadinene | 1.2 | 1.2 | - | 0.1 | 0.1 | 0.1 | 0.2 | 0.1 | 0.1 | 0.9 | - | - | 0.1 |
| Cubebol | - | - | - | - | - | - | - | - | - | - | - | - | - |
| Germacrene-d-4-ol | - | - | - | - | - | - | - | - | - | - | - | - | - |
| Spathulenol | - | 0.1 | - | - | - | - | - | - | - | 0.1 | - | - | - |
| τ-Cadinol | - | - | - | - | - | - | - | - | - | - | - | - | - |
| τ-Muurolol | - | - | - | - | - | - | - | - | - | - | - | - | - |
| α-Cadinol | - | 0.1 | - | - | - | - | - | - | - | - | - | - | - |

**Table A3.** Chemical composition (%, percentages of the total EO composition) and enantiomeric excess of (±)-α-pinene of commercial EOs of *P. sylvestris* (**1**–**11**) and turpentine oils (**12**–**13**); -: not detected.

| **Compounds** | **1** | **2** | **3** | **4** | **5** | **6** | **7** | **8** | **9** | **10** | **11** | **12** | **13** |
| --- | --- | --- | --- | --- | --- | --- | --- | --- | --- | --- | --- | --- | --- |
| Oplapanone | - | - | **-** | **-** | **-** | **-** | **-** | **-** | **-** | **-** | **-** | **-** | - |
| Manool oxide | - | - | - | - | - | - | - | - | - | - | - | - | - |
| Isoabienol | - | - | - | - | - | - | - | - | - | - | - | - | - |
| Sandaracopimaral | - | - | - | - | - | - | - | - | - | - | - | - | - |
| *cis*-Abienol | - | - | - | - | - | - | - | - | - | - | - | - | - |
| Palustral | - | - | - | - | - | - | - | - | - | - | - | - | - |
| Isopimaral | - | - | - | - | - | - | - | - | - | - | - | - | - |
| **Enantiomeric ratio** |  | | | | | | | | | | | | |
| (+)-α-Pinene | 1 | 1 | 1 | 1 | 1 | 1 | 1 | 1 | 1 | 1 | 1 | 1 | 1 |
| (–)-α-Pinene | 1.1 | 1.5 | 8.2 | 7.3 | 6.8 | 10.3 | 5.5 | 7.4 | 5.0 | 1.2 | 10.8 | 8.0 | 12.6 |
| **Enantiomeric excess** |  | | | | | | | | | | | | |
| (+)-α-Pinene | - | - | - | - | - | - | - | - | - | - | - | - | - |
| (–)-α-Pinene | 3.1 ± 0.0 | 19.8 ± 0.0 | 78.4 ± 0.1 | 76.0 ± 0.0 | 74.3 ± 0.0 | 82.4 ± 0.0 | 69.1 ± 0.0 | 76.1 ± 0.1 | 66.7 ± 0.1 | 8.6 ± 0.1 | 83.0 ± 0.0 | 77.9 ± 0.0 | 85.3 ± 0.0 |

**Table A3.** (continued).

| **EO** | **14** | **15** | **16** | **17** | **18** | **19** | **20** | **21** | **22** | **23** | **24** | **25** | **26** | **27** |
| --- | --- | --- | --- | --- | --- | --- | --- | --- | --- | --- | --- | --- | --- | --- |
| **Enantiomeric ratio** |  | | | | | | | | | | | | | |
| (+)-α-Pinene | 2.0 | 4.3 | 2.7 | 7.2 | 3.1 | 1 | 1 | 4.8 | 1.7 | 3.2 | 1.6 | 6.7 | 2.0 | 2.4 |
| (–)-α-Pinene | 1 | 1 | 1 | 1 | 1 | 1.0 | 1.1 | 1 | 1 | 1 | 1 | 1 | 1 | 1 |
| **Enantiomeric excess** |  | | | | | | | | | | | | | |
| (+)-α-Pinene | 34.0 ± 1.4 | 62.0 ± 0.9 | 45.6 ± 1.2 | 75.5 ± 0.3 | 51.7 ± 0.2 | - | - | 65.8 ± 0.4 | 25.8 ± 0.3 | 52.1 ± 0.5 | 23.0 ± 0.2 | 74.0 ± 0.1 | 34.0 ± 0.1 | 40.4 ± 0.1 |
| (–)-α-Pinene | - | - | - | - | - | 0.8 ± 0.7 | 3.2 ± 0.1 | - | - | - | - | - | - | - |
| **EO** | **28** | **29** | **30** | **31** | **32** | **33** | **34** | **35** | **36** | **37** | **38** | **39** | **40** | **41** |
| **Enantiomeric ratio** |  | | | | | | | | | | | | | |
| (+)-α-Pinene | 2.2 | 3.0 | 2.3 | 1.5 | 1.4 | 1.3 | 1.8 | 1.3 | 2.7 | 4.4 | 3.2 | 2.4 | 1 | 6.8 |
| (–)-α-Pinene | 1 | 1 | 1 | 1 | 1 | 1 | 1 | 1 | 1 | 1 | 1 | 1 | 1.4 | 1 |
| **Enantiomeric excess** |  | | | | | | | | | | | | | |
| (+)-α-Pinene | 38.0 ± 0.2 | 50.3 ± 0.0 | 39.4 ± 0.0 | 19.1 ± 0.2 | 17.7 ± 0.1 | 11.4 ± 0.0 | 27.9 ± 0.0 | 13.6 ± 0.1 | 46.6 ± 0.2 | 63.1 ± 0.3 | 52.2 ± 0.1 | 41.9 ± 0.3 | - | 74.3 ± 0.1 |
| (–)-α-Pinene | - | - | - | - | - | - | - | - | - | - | - | - | 15.1 ± 0.1 | - |
| **EO** | **42** | **43** | **44** | **45** | **46** | **47** | **48** | **49** | **50** | **51** | **52** | **53** | **54** | **55** |
| **Enantiomeric ratio** |  | | | | | | | | | | | | | |
| (+)-α-Pinene | 6.6 | 9.6 | 6.8 | 8.8 | 7.5 | 1 | 1 | 1 | 1 | 1 | 1 | 1 | 1.1 | 1 |
| (–)-α-Pinene | 1 | 1 | 1 | 1 | 1 | 4.8 | 1.1 | 3.5 | 1.7 | 1.4 | 1.9 | 2.6 | 1 | 5.0 |
| **Enantiomeric excess** |  | | | | | | | | | | | | | |
| (+)-α-Pinene | 73.6 ± 0.1 | 81.2 ± 0.0 | 74.3 ± 0.0 | 79.6 ± 0.1 | 76.4 ± 0.2 | - | - | - | - | - | - | - | 5.2 ± 0.1 | - |
| (–)-α-Pinene | - | - | - | - | - | 65.2 ± 0.4 | 3.8 ± 1.9 | 55.8 ± 0.4 | 25.8 ± 0.1 | 18.2 ± 1.0 | 30.4 ± 0.4 | 43.9 ± 0.5 | - | 66.5 ± 0.3 |
| **EO** | **56** | **57** | **58** | **59** | **60** | **61** | **62** | **63** | **64** | **65** | **66** | **67** |  |  |
| **Enantiomeric ratio** |  | | | | | | | | | | | | | |
| (+)-α-Pinene | 1 | 1 | 1 | 1 | 13.0 | 11.0 | 11.5 | 11.7 | 7.4 | 4.9 | 5.6 | 5.2 |  |  |
| (–)-α-Pinene | 18.0 | 23.6 | 3.1 | 8.3 | 1 | 1 | 1 | 1 | 1 | 1 | 1 | 1 |  |  |
| **Enantiomeric excess** |  | | | | | | | | | | | | | |
| (+)-α-Pinene | - | - | - | - | 85.7 ± 0.1 | 83.4 ± 0.1 | 84.0 ± 0.1 | 84.2 ± 0.1 | 76.1 ± 0.1 | 65.9 ± 0.1 | 69.6 ± 0.2 | 67.8 ± 0.1 |  |  |
| (–)-α-Pinene | 89.5 ± 0.0 | 91.9 ± 0.2 | 51.6 ± 0.1 | 78.4 ± 0.2 | - | - | - | - | - | - | - | - |  |  |

**Table A4:** Enantiomeric ratio and excess of (±)-α-pinene of primary EOs of *P. sylvestris* (**14**–**40**), *P. cembra* (**41**–**46**), *P. mugo* (**47**–**54**), *P. nigra* (**55**–**59**) and *P. sibirica* (**60**–**67**); -: not detected.


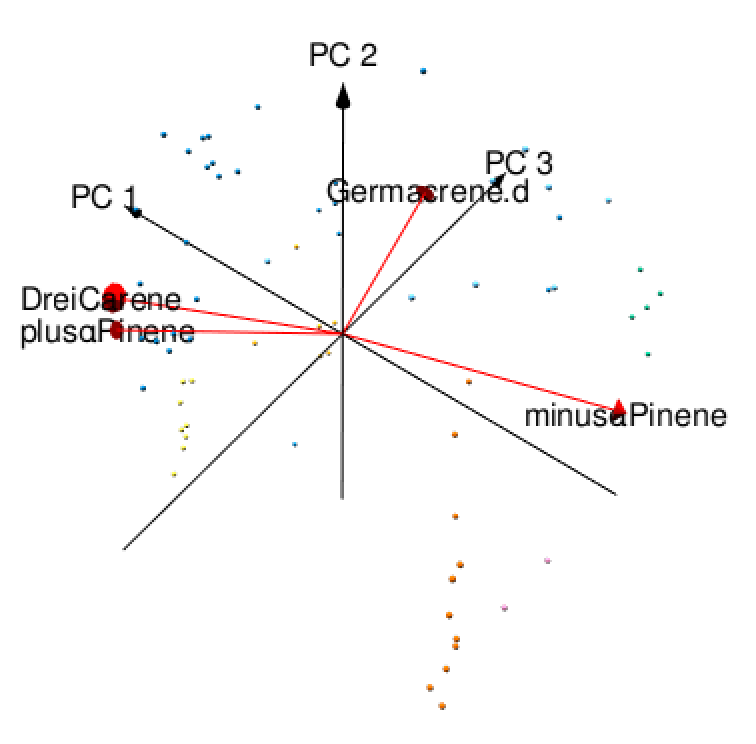


**Figure A1.** 3D biplot of PC1, PC2 and PC3 for primary pine, commercial EOs and turpentine oils based on their chemical composition obtained by the conventional GC-FID and (±)-α-pinene.
